# Health Problems and Risk Factors Associated with Long Haul Transport of Horses in Australia

**DOI:** 10.3390/ani5040412

**Published:** 2015-12-10

**Authors:** Barbara Padalino, Evelyn Hall, Sharanne Raidal, Pietro Celi, Peter Knight, Leo Jeffcott, Gary Muscatello

**Affiliations:** 1 The Faculty of Veterinary Science, The University of Sydney, 425 Werombi Road, Camden 2570, NSW, Australia; E-Mails: evelyn.hall@sydney.edu.au (E.H.); leo.jeffcott@sydney.edu.au (L.J.); gary.muscatello@sydney.edu.au (G.M.); 2 Department of Veterinary Medicine, The University of Bari, Bari 70100, Italy; 3 School of Animal and Veterinary Science, Charles Stuart University, Wagga Wagga 2650, NSW, Australia; E-Mail: sraidal@csu.edu.au; 4 DSM Nutritional Products, Animal Nutrition and Health, Columbia, MD 21045, USA; E-Mail: pietro.celi@dsm.com; 5 Faculty of Veterinary and Agricultural Sciences, The University of Melbourne, Parkville 3010, Australia; 6 Discipline of Biomedical Science, School of Medical Sciences, Sydney Medical School, University of Sydney, Lidcombe 1825, Australia; E-Mail: peter.knight@sydney.edu.au

**Keywords:** transport, horse, journey duration, season, risk

## Abstract

**Simple Summary:**

Records from road transport of horses from Perth to Sydney over a two year period were analysed to explore the incidence of transport related issues and identify risk factors. Transportation resulted in health problems in 2.8% of the transported horses, and in fatalities in 0.24%. Journey duration and season were risk factors for the development of transport related health problems, while breed, sex and age did not predict disease or injury risk. Overall, this study provides statistics to inform policy development for the equine transport industry and enhance management of the transported horse.

**Abstract:**

Equine transportation is associated with a variety of serious health disorders causing economic losses. However; statistics on horse transport are limited and epidemiological data on transport related diseases are available only for horses transported to abattoirs for slaughter. This study analysed reports of transport related health problems identified by drivers and horse owners for 180 journeys of an Australian horse transport company transporting horses between Perth and Sydney (~4000 km) in 2013–2015. Records showed that 97.2% (1604/1650) of the horses arrived at their destination with no clinical signs of disease or injury. Based on the veterinary reports of the affected horses; the most common issues were respiratory problems (27%); gastrointestinal problems (27%); pyrexia (19%); traumatic injuries (15%); and death (12%). Journey duration and season had a significant effect on the distribution of transport related issues (*p* < 0.05); with a marked increase of the proportion of the most severe problems (*i.e.*, gastrointestinal; respiratory problems and death) in spring and after 20 h in transit. Although not statistically significant; elevated disease rate predictions were seen for stallions/colts; horses aged over 10 years; and Thoroughbreds. Overall; the data demonstrate that long haul transportation is a risk for horse health and welfare and requires appropriate management to minimize transport stress.

## 1. Introduction

Transport stress in horses is caused by a myriad of stressors (e.g., isolation, confinement, noise, vibration, balance problem) which affect them both mentally and physically, causing behavioural and health problems prior to, during and after travel [1]. Both short and long trips are stressful for horses and require proper management [2]. Longer trips have a greater effect on horse health and require particular attention [3,4], and those longer than 10 h duration may lead to psychological and physical exhaustion and death [5]. Consequently, many animal transport codes include special requirements for longer journeys. For instance, the EU regulation 1/2005 [6] has specific requirements if the transport exceeds 8 h. The Australian Animal Welfare Standards and Guidelines for the Land Transport of Livestock has instead specie-specific maximum journey and minimum rest periods that take into account access to water and food en route [7].

Stress activates hormonal changes in animals, which help them to adapt to the stressful situation. This response is commonly referred as “the flight or fight response”, and it is characterized by the activation of the pituitary and adrenal responses and by a release of adrenaline and cortisol. The most common effects of adrenaline are an increase in heart and respiratory rates, and an increase in sweating and defecation [8]. During transportation these hormonal responses are often a result of the horse attempting to adapt to the challenging situation (being transported), but they can affect the horse’s immune response, making the horse more susceptible to transport-related diseases [9,10].

Transportation has been associated with physical injuries and heat stress, as well as specific illnesses such as respiratory diseases, colic, laminitis, enterocolitis and rhabdomyolsis [1,3,11]. The most serious, potentially fatal respiratory disease is equine pleuropneumonia, commonly referred to as “travel sickness” or “shipping fever” [12]. The risk of developing this disease increases with journey duration, especially when the duration exceeds 10 h [13]. Predisposing factors for the development of shipping fever include prolonged head elevation [14], poor air quality [15], and pre-existing respiratory diseases [5]. Transport associated dehydration, withholding of food and water, and diet change on arrival have been proposed as risk factors in the development of transport related gastrointestinal disease in horses [1]. Colic during or after transportation is commonly reported, with impaction of large colon most often recognised [1,11]. Enterocolitis caused by *Salmonella spp*. has been also associated with transport stress [16] and can be fatal. While many risk factors for the development of transport related diseases and injuries have been identified, further studies are required to identify additional unrecognised factors, and to determine the relative contribution of different contributing factors to transport related disease and injury. Knowledge of the full range of risk factors related to equine transportation may help to safeguard the welfare and wellbeing of horses.

Surveys on farm animal transportation have been performed in to identify risk factors and explore epidemiological basis of transport related health and welfare issues. For instance, the incidence of mortality during road transport has been calculated for cattle in North America (0.011%) [17], bobby calves in Australia (0.64%) [18], pigs in Europe (0.07%) [19] and in broilers in Brazil (from 0.42% in summer to 0.23% in autumn) [20]. In horses, surveys have been reported only for transport to abattoirs/slaughter plants [21,22,23]. In these studies, transport related health problems ranged from 7% to 28%. However, large numbers of horses are transported for other commercial activities such as competition and breeding, and for recreational uses. As these animals have a greater economic value than those destined for abattoirs, it is likely that their management and their transport-related health problem incidence will be different.

Millions of horses are moved daily all over the world, with the true global total of horse transport movements so large that it is impossible to estimate [24]. Consequently, there is a gap in our knowledge of the incidence of transport related problems, horse mortality, and risk assessment related to equine commercial transportation. To the authors’ knowledge, a survey on commercial equine road transport for any purpose has never been conducted in Australia. As the scientific identification and evaluation of hazards can only be done when the scenario including the animal and the transport environment is defined [25], the records of a horse transport company specialized in long road trips (~4000 km, taking 3.5 days) in Australia were collected and analysed. The objective of the present study was to determine the incidence of transport related injury and illness in horses undertaking commercial long-distance road transportation, and to assess and quantify the relationship between animal (sex, breed, and age), transport conditions (duration, and season) and welfare outcomes measured by the incidence of death, injuries, pyrexia, respiratory and gastrointestinal problems associated with a defined commercial long haul transport in Australia.

## 2. Experimental Section

### 2.1. Materials and Methods

Records of all transport movements from April 2013 to April 2015 were obtained from a commercial horse transport company which regularly transports horses between the east and west coast of Australia (~4000 km and at least 3.5 days duration). Care and handling of the animals during transportation was not supervised by the research team. This data set was collected as part of a comprehensive survey on horse transportation approved by the Human Research Ethics Committee of the University of Sydney (2015/308).

### 2.2. Trip Details

Before booking the trip, each owner had to send to the company the following information: breed, sex, age, body measurement, level of tame, reason for transportation. This information was necessary to allocate the right space to each animal inside the vehicle. As policy the company moved only tamed horses, at least trained to halter and basic commands from ground (e.g., follow and stop at the rope) and advised previous transport experience, the transported animals complied with this policy.

All transportation was performed following a fixed schedule from a collection stable in Sydney. The trip consisted of four stages: Sydney-Melbourne (10 h), Melbourne-Adelaide (8.5 h), Adelaide-Kalgoorlie (24 h) and Kalgoorlie-Perth (6 h). After each stage, horses were given a twelve hour rest period. The total duration was approximately 85 h with approximately 49 h in transit and 36 h for rest stops. The schedule was reversed for Perth-Sydney trips.

At the collection stable and rest points, horses were individually housed in in-walk out rubber lined stables and/or paddocks that were used only for horses in transit.

All animals travelled on the same type of vehicle (Mega Ark Trailers, MAN^®^, Munich, Germany, Europe) equipped with 15 horse individual stalls, 6 facing backwards and 9 facing forwards. However, since large horses were allocated 1½ stall spaces, the average number of horses transported per trip was 9.1.

The ventilation system comprised venturi vents, louvres and electric fans generating an airflow which the manufacturer verified was compliant with the Australian code of transportation throughout the trailer. When the vehicle was moving fresh air entered through the louvres and was extracted by the venturi vents. The fans were used in extreme heat conditions (> 35 °C–40 °C), and to ensure constant air flow when the truck was stationary (e.g., feeding and watering times, fuel stops).

The horses travelled in individual stalls, restrained by rubber cords which would break under extreme pressure. Foals, ponies, weanlings or un-educated horses were not tied up. Mares and foals travelled in a 3 stall section which allowed them to move around as if in a small box. Horses were fed and watered every 4–5 h, using the stainless steel feed and water bins in each bay; water and food were refreshed regularly en route.

Two drivers were used for all trips for which data were collected. Both were licensed to drive heavy combination vehicles and were experienced horse handlers with many years’ experience in commercial horse enterprises.

All journeys complied with the standards and the guidelines for the transport of horses required by the Australian Code of Livestock Land Transportation [26].

### 2.3. Monitoring of the Animals and Identification of Pathology

The assessment of the fitness for travel of the horses was performed by the drivers and experienced staff members of the company at the collection stable and at each transit stable before loading the animals to continue the journey. The assessment was made in accordance with the Australian Code of Livestock Land Transportation. The condition to be assessed included any signs of colic, raised or lowered rectal temperature, lethargy, diarrhoea, wound or abscesses, lameness (no more or equal to the fourth grade) and body condition score (no less of two) [26]. After the assessment the report was sent to the operation manager, who gave final approval for transport.

During transport, there were two opportunities for monitoring horse health. The first was during the mandatory rest stops which are required after four hours of driving. At these stops, the driver undertook a visual examination of the horses. The second opportunity for monitoring occurred at the transit stables where rectal temperature was recorded, and drinking, feeding and eliminating behaviours monitored. At the rest stop in Adelaide, horses were inspected by an Australian government accredited veterinarian who confirmed that horses were fit to continue their journey, administered a triclabendazole drench and collected a faecal sample in compliance with western Australian quarantine regulations.

As soon as any health problem was identified by the drivers, the company manager was informed and a veterinarian was called for consultation and for treating the affected horse. The company director had a list of veterinarians to call in emergency. The affected horse did not continue the trip if the veterinarian did not evaluate it fit for travel. When health problems were identified by the owners post transport, the transport company manager was informed and he required a veterinarian’s report to prove that the problem was related to the previous journey.

### 2.4. Dataset

The data set included 1650 horses transported from Perth to Sydney (~4000 km) or vice versa for 180 journeys. Horse details (breed, sex, age) and the date of the trip (month and year) were recorded. The data set included reports of problems and issues identified by the drivers and horse owners and sent to the company manager, including the type of problem(s) and where (e.g., location) and when it occurred (*i.e.*, an estimation of the approximate time at which the issues or incidents were first identified). As after the identification of each problem, horses were checked and treated by veterinarians, and after death necroscopy was conducted, their veterinary records were also included in the dataset.

For statistical analysis, the recorded transport related issues and problems were classified according to the time of occurrence in the following categories: pre-loading (from the horse’s home stable to the company’s collection stable in Sydney or Perth); in transit (during the trip or at rest stops); and post-transport (within 3 days after arrival at destination).

Based on the veterinary records, the transport related health problems were also classified into five categories according to clinical signs/body system affected (Table 1).

**Table 1:** Description of the transport related issues.

Category	Definition
Injuries	Laceration, abrasion, contusion, swelling.
Pyrexia	Rectal temperature >38.5 °C, in the absence of other localising signs.
Gastrointestinal problems	Colic, enterocolitis, large quantity of internal parasites eliminated after triclabendazole treatment.
Respiratory problems	Nasal discharge, coughing, inflammation/infection of the upper or lower respiratory tract, and pneumonia.
Death	Horses found dead or humanely destroyed.

### 2.5. Statistical Analysis

Descriptive analysis of the dataset was conducted using statulor^beta^ [27]; data were reported as number of injuries or illnesses and as percentages. All further statistical analyses were performed using Gen Stat^®^ Version 14 (VSNi International, Hemel Hempstead, UK). For all statistical analyses, a *p* value of <0.05 was considered statistically significant.

The details of all the travelled horses were categorized according to sex (mare/filly, gelding, stallion/colt), age (weaning/foal, yearling, 2–5 years, 6–10 years, >10 years), and breed (Arab, Quarter horse, Standardbred, Thoroughbred, Warm Blood). Univariate logistic regression analysis was conducted with development of a transport-related problem as the outcome (1/0: affected/non affected), and sex, breed, and age as the explanatory variables. Wald tests were obtained along with mean predictions of disease rate for each variable.

The date of recorded transport-related issues was categorized into the four Southern hemisphere seasons: winter (June–August), spring (September–November), summer (December–February), autumn (March–May). The time of the recorded transport-related issues (calculated from departure to when the recorded transport related issue or problem was identified) was classified into three categories of journey duration: <20 h, 21 h–40 h, and >40 h. Based on the veterinary records, considering the severity of the clinical signs, the required treatments and the time of recovery, the type of transport-related issues was listed in order of increasing severity as follow: injuries, pyrexia, gastrointestinal problems, respiratory problems, death. Ordinal regression analysis was then conducted to study the association between the type of transport-related issues (outcome) and the journey duration (<20 h, 21 h–40 h, >40 h), and the season of the year (winter, autumn, summer, spring) (factors).

## 3. Results

### 3.1. Descriptive Statistics

The general demographics of the population of horses studied is shown in Table 2. Horses were transported for the following reasons: sales-purchase (30%), competition (50%), and breeding (20%).

**Table 2:** Frequency of the total transported horses by sex, breed and age category.

Variable	Category	Frequency (%)
Sex	Gelding	35.7
	Mare/Filly	49.5
	Stallion/Colt	14.8
Breed	Arab	9.6
	Quarter horse	8.7
	Standardbred	27.5
	Thoroughbred	43.0
	Warm blood	11.2
Age	Weaning/Foal	11.2
	Yearling	12.9
	2–5 yrs	34.7
	6–10 yrs	27.9
	>10 yrs	13.3

Approximately 97 % (1604/1650) of the horses arrived at destination in good health, without any pain, signs of lameness or other pathology and did not develop any diseases post journey.

**Figure 1:**
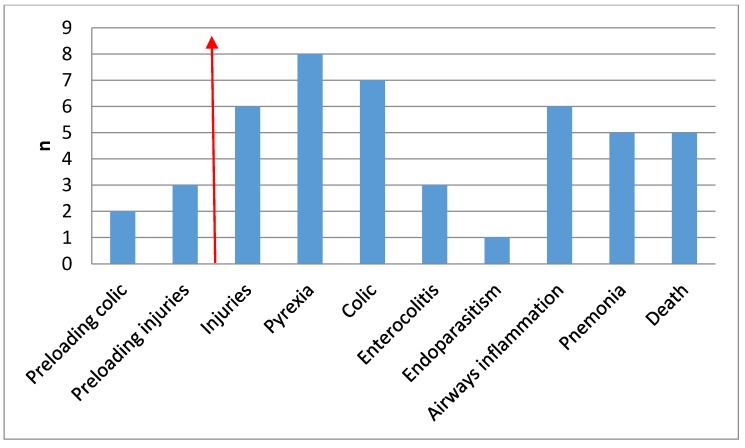
Incidence of transport-related issues as reported by the transport company. The arrow divides issues related to pre-loading from those related to transit and post transit phase.

Only 2.8% (46/1650) were included in the company dataset for a transport related issue at pre-loading, in transit, or post-transit (Figure 1). Of the 46 cases, five cases related to pre-loading events, three were injuries that had occurred before the trip commenced or during transport to the collection stable, and two were cases of colic identified at the departure stable. Of the remaining cases, two horses were injured during loading while resisting loading and 4 injuries happened in transit. All the injuries were minor and the horses were treated topically and continued their journey. Six horses were identified as febrile at rest stops, and another two were identified as febrile upon arrival. No localising signs were identified in any of these courses and all were treated with anti-inflammatory medications. Four horses showed signs of colic at rest stops with another three showing signs of colic post journey. All cases were interpreted as impaction colic; two resolved without treatment (required only monitoring) and 5 were treated medically. Enterocolitis was identified in one horse during transport, and in two horses post transport, with all horses requiring hospitalisation. One horse eliminated a massive quantity of parasites after the anti-parasite treatment. Five horses developed respiratory signs, including nasal discharge, coughing, and pyrexia during the journey, and one developed signs after arrival. The veterinary diagnosis was inflammation of the upper or lower airways, without pneumonia and all cases were treated medically. The specific diagnosis of pneumonia was made on the basis of signs that developed in four horses during transport, and in one horse after arrival. All horses recovered after appropriate medical treatment. There were four transport related deaths, giving an overall death rate of 0.24%. Two occurred during transport, one horse was found dead within 24 h after transportation, and one was humanely destroyed due to enterocolitis post transit. Another horse was found dead two days after transport, and it cannot be confirmed that the death was transport related. Post-mortem examination failed to reveal the cause of death in the four horses that were found dead. If the horse that was found dead two days after transport is included in the statistics, the death rate increases to 0.30%.

The incidence of the transport-related issues grouped by category is shown in Table 3.

**Table 3:** Incidence of transportation issues grouped in 5 major categories according to clinical signs and body system affected.

Category	*n*	Incidence on the Affected Animals (*n* = 41)	Incidence on All Transported Animals (*n* = 1650)
Injuries	6	15%	0.36%
Pyrexia	8	19%	0.48%
Gastrointestinal problems	11	27%	0.66%
Respiratory problems	11	27%	0.66%
Death	5	12%	0.30%

### 3.2. Logistic Regression

Univariate logistic regression analysis of horses experiencing transport related health issues showed no significant effect of sex, breed, or age (Table 4).

**Table 4:** Results of the univariate logistic regression analysis with development of a transport-related problem as the outcome (1/0: affected/non affected), with sex, breed, and age as explanatory variable.

Variable	Category	Disease Rate Prediction (%) ± s.e	Estimate ± s.e (%)	OR	Lower 5% CI	Upper 95% CI	*p* Value
Sex	Gelding	7.0 ± 1.5	Ref.				0.611
	Mare/Filly	5.3 ± 1.1	−0.29 ± 0.3	0.74	0.39	1.424	
	Stallion/Colt	7.1 ± 2.4	0.02 ± 0.4	1.02	0.43	2.403	
Breed	Arab	3.3 ± 2.2	Ref.				0.187
	Quarter horse	5.4 ± 3.0	0.14 ± 0.7	1.16	0.25	5.217	
	Standardbred	5.2 ± 1.6	0.56 ± 0.6	1.75	0.50	6.039	
	Thoroughbred	9.2 ± 1.7	0.72 ± 0.6	2.05	0.59	7.141	
	Warm blood	2.5 ± 1.9	0.96 ± 0.6	2.61	0.70	9.743	
Age	Weaning/Foal	1.8 ± 1.0	Ref.				0.523
	Yearling	2.1 ± 1.0	0.51 ± 0.9	1.67	0.26	10.41	
	2–5 yrs	3.2 ± 0.8	−7.20 ± 9.3	0.00	8.12E-12	68858	
	6–10 yrs	3.8 ± 0.9	1.08 ± 0.7	2.94	0.67	12.8	
	>10 yrs	4.8 ± 1.5	−0.10 ± 1.0	0.85	0.11	6.246	

Standard error (s.e), Odds ratio (OR), Confidence Interval (CI).

### 3.3. Ordinal Regression Analysis

**Figure 2:**
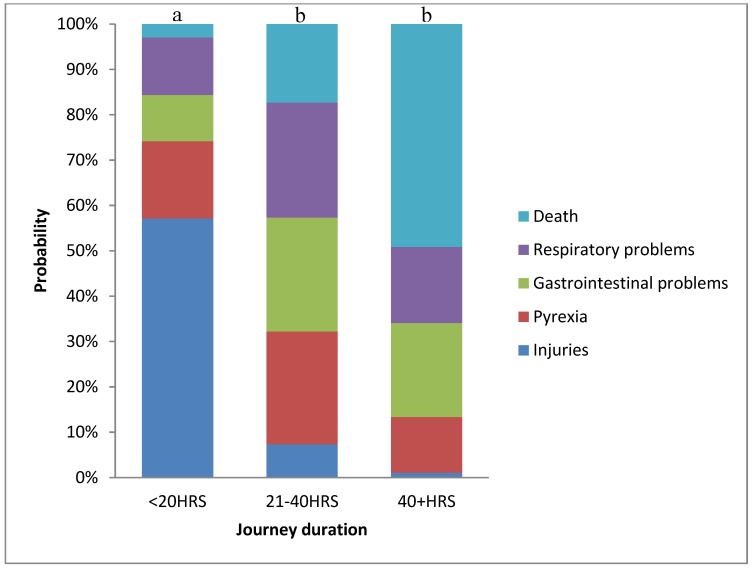
Probability of a more severe transport-related issue to be associated with journey-duration. Bar charts with different letter have a different distribution of transport-related issues: a, b: *p* < 0.05.

There was a significant association (*p* = 0.022) between type of transport related issues and duration of trip, with a higher probability of a more severe disease after 20 h of transport. Injuries were more likely to occur in the first 20 h of transport (Figure 2).

Table 5 shows odd ratio and confidence interval for each disease occurring in a journey longer than 20 h.

**Table 5:** Estimate, odds ratio (OR) and confidence interval (CI) for each transport-related problem on a journey longer than 20 h.

Transport-Related Problem	Estimate	s.e	*p* Value	OR	Lower 95% CI OR	Upper 95% CI OR
Injury	-	-	-	-	-	-
Fever	2.56	1.34	0.057	12.91	0.9271	179.8
Colic	3.19	1.3	0.014	24.37	1.91	311.1
Respiratory	2.93	1.32	0.027	18.69	1.399	249.7
Death	4.54	1.52	0.003	93.49	4.783	1827

**Figure 3:**
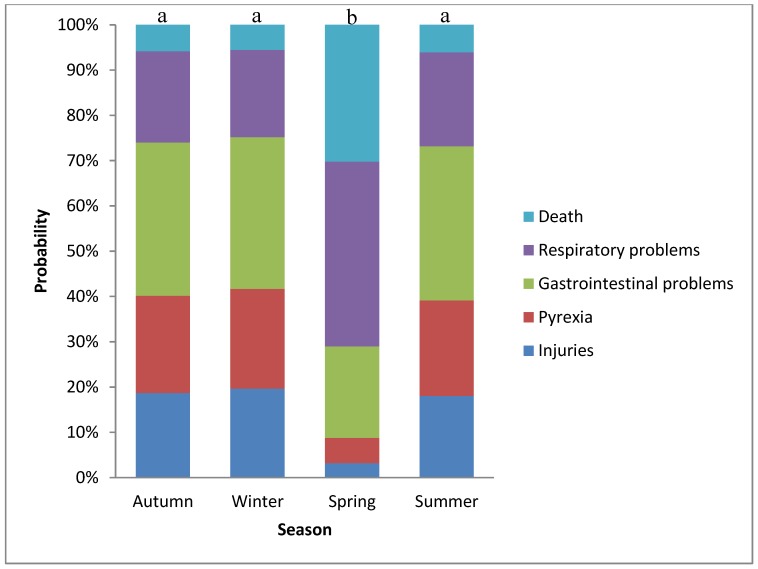
Probability of a more severe transport-related issue to be associated with season. Bar charts with different letter have a different distribution of transport-related issues: a, b: *p* < 0.05

Season had a significant effect on the distribution of transport-related issues (*p* = 0.035), with a higher probability to have a more severe transport-related issue (gastrointestinal problems, respiratory problems and death) in spring (Figure 3).

## 4. Discussion

The present study reports the incidence of transport-related issues and mortality associated with long-haul equine transport by a commercial equine transport company. An overall incidence of transport related injuries or disease of 2.8% was observed in this study, which is much lower than has been reported by horse owners [28,29] and for horses transported to abattoirs for slaughter [21,23]. The data demonstrates that travelling is a risk to equine health and welfare and a correct management of transportation is required for moving horse successfully.

The prevalence of injuries identified in the present study (0.36% of horses) was lower than the rate reported in horses transported to abattoir for slaughter [21,23]. This may reflect differences in the way in which the transport was managed (*i.e.*, individual calculated space), and in the tractability and transport experience of the horses. However, it was also lower than the reported by owners during non-commercial horse transportation [29], which suggests that transport management is a key determinant of the injury rate. The design of the truck (including the floor, suspension and the height of height of the roof) has been identified as a risk factor in the development of injuries and transport related diseases in farm animals [25]. In cattle, the incidence of injuries during long haul transportation has been associated with the years of experience of the drivers [17], and in horses travelling on non-commercial road transport, many incidents are related to poor driving skills, particularly on winding country roads [29]. Vehicle design, road quality and driving that allows horses to keep their balance appears to be important in minimising injuries.

Two injuries occurred during the loading process due to resistance to boarding the truck. The horses’ fear of being loaded onto the vehicle manifests through various deleterious and dangerous actions and behaviours (e.g., kicking, rearing) [1]. Handling horses during loading/unloading is therefore considered to be highly dangerous risk for those handling the transported horse [30]. Thus it is important that transport procedures are carried out by experienced horse handlers wearing protective equipment, such as, capped boots and gloves, to minimize the risk of injuries to the horse and the handler during the loading and unloading phase of equine transport.

The body systems most commonly affected by transport are the respiratory and the gastrointestinal systems, and a common clinical sign associated with inflammation to these systems is pyrexia [5]. Pyrexia affected 0.48% of all transported horses in this study. Since early identification of pyrexia prompts investigation and implementation of appropriate therapy and recovery, checking temperature during and after long trips should be seen as best practice when dealing with the transported horse and has been recommended previously [31,32].

Transportation has been associated with the development of airway inflammation and equine pleuro-pneumonia [12]. Poor ventilation inside the truck, forced high head position, and dehydration have been identified as predisposition factors in the development of respiratory diseases associated with transport [3]. The horses in the current study travelled in a vehicle equipped with a forced ventilation system, which should assure good air quality and a comfortable temperature inside the trailer, but horses were not allowed to lower their head beyond the height of the wither. In our study 0.66% of all transported horses developed respiratory problems, and only five developed pneumonia. This rate is less than expected [28], potentially reflecting the importance of a good ventilation system in the transport vehicle. However, there is no evidence to suggest that good ventilation alleviates the need to provide correct watering [33], to minimise the duration of confinement with the head elevated, or to maximize the time available for the horse to physically clear its respiratory tract [34].

Transportation may increase the likelihood of colic for several reasons [35]. Firstly during a stressful situation, preferential perfusion of the brain and the muscles may reduce visceral perfusions (flight and fight response) [8]. Additionally, dehydration during transportation can reduce vascular perfusion of the gut, potentially inducing impaction of the colon [11]. Finally food and water withdrawal, altered diet and/or eating in an unnatural position on route might create change in the pH and gut flora which may influence the chance of colitis. In this study, transport associated gastrointestinal problems were seen in 27% of cases, with enterocolitis seen in 4 out 41 cases, with one requiring euthanasia. Equine enterocolitis, can manifest in sudden death and be associated with over-proliferation of *Salmonella spp*, *Clostridium spp* and *Fusarium spp* in the equine gut [36]. Stress is considered an important predisposing factor for salmonellosis in horses; this pathology has already been associated with transportation, surgery, feed withdrawal, changes in feed, and antimicrobial and anthelminthic therapy [16]. Avoiding prolonged feed and water restriction, abrupt diet changes, or overuse of antibiotics and anthelminthics before, during and after transportation could therefore potentially reduce the incidence of gastrointestinal transport associated illness.

In this study the mortality rate associated with transport events was 0.24% or 0.30%, which is somewhat higher than rates reported for cattle in North America (0.01%) [17] and pigs in Europe (0.07%) [19]. A reason could be that animal transportation is more risky in Australia due to the long journey duration and the climate; the rate described in this study is indeed in line with mortality rates observed in cattle transported in Queensland by rail (an overall mortality rate of 0.10%, ranging from 0.44% in bulls to 0.06% in calves) [37], and in bobby calves (>4 days old) transported by road in Victoria (0.64%) [18]. Thus, moving horses in Australia may require more detailed and specific strategies to cope with extreme distance and weather.

In the current study, two horses were found dead in transit and two died soon after transportation. Even after pathological examination the reasons for these deaths were unknown. It is possible that protracted stress may have contributed to death. Stress is a physiological and endocrionological response that helps individuals to cope with stressors and to survive. However, when an animal fails to adapt, the stress response can lead to death [8]. In horses, transport stress is often followed by the stress of living in unfamiliar environments (e.g., new stall, food, social group), further affecting the horse’s health. Consequently, offering similar feed and avoid inserting the recent arrival into a new herd could reduce protracted stress and assist with adaptation to the unfamiliar environment after the journey [1] and potentially reduce the risk of death after transportation events.

Witnessing a death of any animal can have a negative impact on those who have witnessed it, whether they are professionals working in the animal or veterinary industry or members of the general public owning animals [38]. Consequently, minimising equine transport associated mortality rate will have a positive impact on the wellbeing of horses and the mental wellbeing of those dealing with the transported horse on a day to day basis.

No significant effect of sex, age, or breed in the development of transport diseases was found, suggesting that individual horse variability and past experience might be more important in influencing the ability of the horse to cope with the transport event [39]. Elevated, but not statistically significant, prediction rate were seen for males, horses aged over 10 years, and Thoroughbreds, consequently dealing with these categories of horses may require specific transport management strategies to reduce the predicted risk of these horses developing transport related complications.

In agreement with Stefancic and Martin [22], although numbers were small, this current study showed transported related death and respiratory diseases were more likely to occur in spring. This may reflect abrupt temperature increases at a time when many horses still have winter coats, with consequent, impaired thermoregulation in transit. Alternatively, animal behaviour and immune system may be affected by the reproductive hormonal profile of the breeding season [40]. Other factors, such as the occurrence of viral respiratory tract infections or increased pollen or other allergens might contribute to increased risk of respiratory disease at this time. Such speculations warrant further research.

The data presented in the current study, confirm the increased risk of mortality and disease in horses associated with longer transport events, with more severe diseases (e.g., enterocolitis, pleuro-pneumonia) and death more commonly observed after 20 h of transportation. Horses travelling to abattoirs for slaughter, were similarly more likely to die after protracted transportation [22]; and in cattle, higher mortality rates have been associated with trips longer than 36 h in Australia [37], and longer than 30 h in Canada [17]. Better understanding of the increasing risk of severe transport related diseases and death with increasing duration of transport may encourage the adoption of more rigorous preventive strategies, such as a veterinary examination, for horses that are going to be transported for more than 20 h.

The biggest limitation of this study is that the assessment of the horse health before, during and after journeys was not performed by the authors. Consequently some transport-related problems might have been missed by the drivers and the owners, and the incidence of the transport-related diseases could be underestimated. This is particularly likely if horse owners did not associate illness with recent transport, failed to recognise minor or subclinical disease, or failed to report minor or major illness to the company. Pleuro-pneumonia and enterocolitis can manifest up to a week after transport [5], and other effects of transportation stress can take up to one month to manifest after the event [41]. Hence it is possible that owners or agents may have failed to associate disease with transportation, or may have failed to detect mild effects on horse health. The data obtained in this current study is also limited by the lack of environmental parameters measured or recorded during journeys. Extreme hot and cold temperatures have been identified as risk factors in long haul transportation of farm animals [42]. Notwithstanding these limitations, this study is the first carried out on horses undertaking this unique multi-day road trip across one of the harshest continents in the world. It has provided important data for the equine industry on the incidence of health problems associated with long haul transportation in the horse. Preliminary evaluation has identified and suggested some predisposing factors associated with transport related health problems which warrant further evaluation to enhance policy and practices relating to transportation of the horse.

## 5. Conclusions

Journey duration and season were identified as risk factors contributing to transport related health problems in horses undergoing long distance road transportation. Although the trips were well organized and complied with or exceeded the requirements of the National Code of Practice for the Transportation of Horses, serious diseases still occurred. Moving horses should be considered as a human-related risk to horses and also a horse-related risk to humans [43], so it should be always carried out by professional and experienced horse handlers and drivers, wearing adequate protective equipment, to reduce the risk of injuries and diseases in both horses and humans. Further research to confirm preliminary conclusions based on this data and to recognize other risk factors for the development of equine transport related issues is needed to assist in improvement of the Australian code of horse transportation.
